# The relationship between self-reported and device-based measurements of physical activity and mental distress among adolescents: results from the fit futures study

**DOI:** 10.1186/s12889-025-23902-x

**Published:** 2025-08-02

**Authors:** Kamilla Rognmo, Ida Marie Opdal, Bjørn Helge Handegård, Alexander Horsch, Kjersti Lillevoll, Anne-Sofie Furberg, Christopher Sivert Nielsen, Bente Morseth

**Affiliations:** 1 Department of Psychology, UiT the Arctic University of Norway, Tromsø, Norway; 2 Department of Health and Nursing Science, UiA – University of Agder, Kristiansand, Norway; 3 Regional Centre for Child and Youth Mental Health and Child Welfare, UiT The Arctic University of Norway, Tromsø, Norway; 4 Department of Computer Sciences, UiT the Arctic University of Norway, Tromsø, Norway; 5 Department of Microbiology and Infection Control, The University Hospital of North Norway, Tromsø, Norway; 6 Faculty of Health and Social Sciences, Molde University College, Molde, Norway; 7 Norwegian Institute of Public Health, Oslo, Norway; 8 School of Sport Sciences, UiT the Arctic University of Norway, Tromsø, Norway

**Keywords:** Mental distress, Depression, Physical activity, Device-based measurements, Adolescents, Accelerometer

## Abstract

**Background:**

The potential for physical activity to prevent or alleviate mental distress among adolescents is unclear, partially due to a lack of studies using objective measurements of physical activity. The purpose of the present study is to investigate the cross-sectional and longitudinal relationship between self-reported and device-based measurements of physical activity and mental distress among adolescents. A second aim is to explore the degree to which the relationship differs according to physical activity measurement method.

**Methods:**

Cross-sectional and longitudinal data from the Norwegian population-based Fit Futures study in 2010-11 and 2012-13 were used. Mean age of the participants was 16.2 years at baseline. Physical activity was measured by self-report and by accelerometer. Mental distress was self-reported. Multiple linear regression analyses were used to analyze the association between physical activity and mental distress, adjusted by demographic, health and life-style variables and peer acceptance.

**Results:**

Using cross-sectional data, self-reported physical activity and objectively measured minutes in moderate to vigorous physical activity were negatively associated with mental distress, up until inclusion of peer acceptance as a covariate in the fully adjusted model. After adjusting for peer acceptance, all but three effects were non-significant. Neither self-reported nor objectively measured physical activity at baseline was significantly related to mental distress at follow-up, after adjusting for baseline mental distress.

**Conclusion:**

Cross-sectionally, both self-reported and objectively measured moderate to vigorous physical activity were significantly related to lower mental distress, but adjusting for peer acceptance rendered the associations mainly non-significant. This finding highlights the important role played by social factors among adolescents, possibly impacting both depressive symptoms and physical activity levels. Physical activity at baseline was not related to mental distress at follow-up, adjusted for baseline mental distress, neither for device-based nor self-reported physical activity.

The prevalence of anxiety and depression in the general population have increased by 32.3% and 33.4%, respectively, since 1990 [1]. During the COVID-19 pandemic, even further increases were seen, and have remained higher compared to levels prior to the pandemic [2]. Similarly, among adolescents in Norway, an increase in the proportion experiencing symptoms of anxiety and depression, particularly among teenage girls has been shown [3]. Preventing the development of the most common mental disorders may not only help the individual at risk, but may also be beneficial from a socioeconomic point of view [4], as anxiety and depression are two of the three most common causes of years lived with disability [1, 5]. Also, experiencing depression in adolescence is a major risk factor for a broad range of mental disorders in adulthood [6]. The potential of physical activity to prevent the onset of new or recurrent anxiety and depression, as well as to improve symptoms of these disorders, has been extensively studied, and the results of studies on adult samples clearly indicate an effect [7, 8]. The evidence among adolescents also points in a similar direction [9–11]. However, the variability in the results of studies among adolescents is much higher; some find a significant effect of physical activity on depression (e.g. [12–17]), while others do not (e.g. [18–24]).

The difference in results concerning adolescents may be related to design and measurement issues of the studies. Studies using a cross-sectional design tend to find a weak to moderate significant relationship between physical activity and depression or anxiety [9, 10]. Cross-sectional studies are, however, not able to shed light upon causal effects, and a significant association between physical activity and anxiety or depression may just as well be explained by lower activity levels among individuals with a high symptom load, rather than a preventive effect of activity. Longitudinal studies can be more informative regarding a potentially preventive effect. While some longitudinal studies find a small, significant effect over time [12–17], others fail to find a significant effect [18–20]. Birkeland and colleagues [25] found that physical activity and depressed mood covaries over time, but that neither causally predicts the other. One possible reason for this lack of agreement in longitudinal studies may be that most studies are normally conducted on relatively healthy samples from the general population, with low levels of mental health problems. Thus, increasing physical activity may not add additional positive mental health effects for most of the sample, meaning that mean level effects may come out as non-significant or small.

Almost all previous studies have used self-reported measurements of physical activity. This is problematic because the validity of self-reported instruments for measuring physical activity is modest, particularly among adolescents. A validity study found that adolescents overestimated time in moderate or vigorous activity compared to device-based measurements [26], but the relative ranking based on self-reported activity shows acceptable validity, indicating that those who report being most active, in general are most active [27, 28]. There is also a discrepancy in the results of studies using device-based measurements and studies using self-reported physical activity, which may indicate that the manner of measurement is of importance for the results. Most of the cross-sectional studies using device-based measurements have failed to find a relationship between physical activity and symptoms of anxiety or depression [29], in contrast to the results of studies based on self-reported physical activity [30]. Only a few longitudinal studies on adolescent samples using device-based measurements have been conducted. Kandola et al. [31], found that higher total physical activity (at 12 and 14 years), minutes in moderate or vigorous physical activity (MVPA) (at 12 years), and light activity (at 12, 14 and 16 years) were related to depression at 18 years, whereas in a study by Toseeb and colleagues [21], minutes in MVPA were not related to depressive symptoms three years later. The degree to which change in physical activity was related to change in depression or mental distress over time was investigated by, respectively, Van Dijk et al. [22] and Opdal et al. [23]. Neither study found evidence of a longitudinal relationship. Bell and colleagues found no significant relationship between physical activity and mental health as measured by the Strength and Difficulties Questionnaire (SDQ) total score over a three year follow-up period [32]. However, total physical activity was related to lower scores on the emotional subscale of the SDQ. The fact that studies using self-reported measurements of physical activity to a larger degree tend to find significant relationships to symptoms of depression or anxiety implies a need for studies using both self-reported and device-based measurements of physical activity in the same sample.

A number of variables may impact either physical activity or anxiety or depression and thus need to be accounted for in the models. Demographic variables such as higher parental socioeconomic status [33], lower age [34, 35] and male sex [34, 35] have been associated with higher levels of physical activity, whereas low socioeconomic status [36], female sex [37] and increasing age [38] are known risk factors of anxiety and depression among adolescents. Higher body mass index (BMI) [35, 39] and smoking [40, 41] have also been shown to be related to physical inactivity and anxiety and depression. The degree to which there is a relationship between alcohol use and physical activity is unclear, as studies on the association shows mixed results [40]. Alcohol use has previously been found to be related to higher levels of anxiety or depression [41].

Peer relationships, such as a feeling of being accepted by peers, may also be of importance to the relationship between physical activity and anxiety or depression. Peer acceptance has been found to be associated with factors influencing physical activity levels [42, 43], as well as anxiety and depression [44]. Peer acceptance may be an intermediary variable in the relationship, and as such, targeted by interventions aiming at preventing anxiety or depression among adolescents. On the other hand, peer acceptance may also be a confounding factor, influencing both physical activity levels and symptoms of anxiety or depression, but not a part of a causal chain linking the factors together. Either way, it is important to establish if including peer acceptance in the model impacts the estimates of physical activity on symptoms of anxiety or depression in a significant manner, as a first step in exploring the role played by peer acceptance.

In the present study, we will use both self-reported and device-based measurements of physical activity. Both light and moderate/vigorous physical activity will be explored using accelerometer, providing the opportunity to see if intensity of activity is of importance. Few studies have had the opportunity to use both device-based and self-reported measurements of physical activity in the same sample. The results may be informative about the reliability of studies using self-reported measurements of physical activity when exploring the relationship to mental distress. If the relationships are similar, greater trust in results based on self-reported physical activity may be reasonable. On the contrary, if the relationships diverge, caution is warranted when interpreting results of studies using self-reported physical activity.

The primary aim of the present study is to investigate the relationship between physical activity, as measured by self-report and accelerometer, and mental distress among adolescents. A secondary aim is to explore whether the relationship differs according to measurement method. The analyses will be conducted in a cross-sectional and longitudinal sample, and in the latter, the direct effect of baseline physical activity on mental distress two years later, adjusted for baseline distress, will be investigated. Symptoms of anxiety or depression are conceptualized as mental distress. We hypothesize that both self-reported and device-based measurements of physical activity will have a positive association with mental distress, but that the association will be stronger for the self-reported measurements. Furthermore, we hypothesize that effects will be stronger in the cross-sectional models than in the longitudinal models.

## Methods

### Sample and design

The present study used data from the Fit Futures Study, a general population study of adolescents attending upper secondary school in the municipalities of Tromsø and Balsfjord, in northern Norway. Fit Futures was conducted in 2010–2011 (T1/baseline) and in 2012–2013 (T2/follow-up). The study consisted of a web-based questionnaire, clinical examinations, and interview by trained research personnel. The present study is based on questionnaire data, clinical examinations (height and weight) and accelerometry. The questionnaire was developed to be used in multiple studies, and may be found at the Fit Futures web page [45]. At T1, all first level upper secondary school students in the two municipalities were invited to participate. In total, 1,117 students were invited, of which 1,038 (92.9%) participated. At T2, all students attending third level upper secondary school, and former participants who had left school, were invited (in total 1,129 individuals) and 870 (77%) participated. In total, 714 individuals participated at both T1 and T2 (63.9% of the original sample), of which 449 (67.9% of the T1 and T2 sample) had complete data on all variables included in the analyzed models. In order to avoid bias and increase the statistical power to detect a clinically meaningful effect, multiple imputations were used to impute missing data. After imputations, 699 participants had complete data on all variables used in the analyses, and thus constitutes the sample analyzed in the inferential analyses of the present study.

### Instruments: focal predictor variables

*Physical activity* (T1) was measured by using device-based methodology and self-report. The ActiGraph GT3X accelerometer was used as a device-based measurement. The participants were instructed to wear the ActiGraph on their dominant hip for 8 days, except when sleeping, showering or swimming. Participants with at least 10 h of wear time for a minimum 4 out of the 8 days were considered to have valid data. ActiLife software, provided by the manufacturer (ActiGraph, LLC, Pensacola, USA), was used to initialize the ActiGraph and to download data in 10-second epochs. The Quality Control & Analysis Tool (QCAT) was used for further data processing. For the analyses, the data was aggregated to epochs of 60 s duration. Non-wear time was identified using the triaxial algorithm described by Hecht et al. [35] as it conforms to previous research definition of non-wear time [36]. In total, 91.6% of the sample wore the accelerometer during winter months, and only 8.4% were measured during spring or summer.

The ActiGraph variables of interest in this study are “minutes in light physical activity (LPA) per valid day” and “minutes in moderate to vigorous physical activity (MVPA) per valid day” based on triaxial cut-points identified by Peterson et al. [46] and validated by Sasaki and colleagues [47]. Definitions of LPA and MVPA are described in detail by Sagelv et al. [48]. Minutes in LPA and minutes in MVPA were transformed into number of 15-minute units, in order to simplify interpretation of the estimates in the regression models and ease comparison to previous studies. The ActiGraph GT3X has been validated against indirect calorimetry, with satisfactory results [49], although some activities are underestimated, such as biking and swimming [50].

Physical activity in leisure time was also self-reported in the questionnaire. One item asked about the weekly *frequency of physical activity*, with response alternatives “never”, “less than once per week”, “once per week”, “2–3 times per week”, “4–6 times per week” and “approximately every day”. Subsequently, the response categories were recoded into three categories: “never or rarely”, “2–3 times per week” and “4 or more times per week”.

*Self-reported hours of weekly physical activity* were assessed by asking the participants to state how many hours they were physically active outside of school for one week. Response options were “none”, “about 30 minutes”, “about 1-1.5 hours”, “about 2–3 hours”, “about 4–6 hours” and “7 hours or more”. Next, the response options were recoded as follows: “none to 30 minutes per week”, “1 to 3 hours per week”, and “4 or more hours per week”. This item has previously been used and validated in an adolescent population [51].

*The intensity of physical activity* was assessed by one item asking the participants how hard the physical activity they do outside of school was. The response categories of the original variable were “not hard at all”, “a bit hard”, “quite hard”, “very hard” and “extremely hard”. The response options were recoded into the following categories: “no exercise or not hard”, “a bit to quite hard” and “very to extremely hard” physical activity.

*Level of leisure time physical activity* was measured by using the Saltin-Grimby Physical Activity Scale (SGPALS) [52]. SGPALS consists of four statements concerning activities that best represent the physical activity level of the individual, from (1) physically inactive, (2) some light physical activity, (3) regular physical activity and training to (4) regular hard physical training and competitive sports. Each of the four levels were exemplified by various activities. SGPALS has been found to have satisfactory ranking validity, in a study based on data from the same sample as the present study [27].

The self-reported items measured physical activity outside school during the past year, whereas the device-based measurements assessed physical activity in both school and leisure time during a one-week period. However, for the purpose of the present study, only the ranking of participants in terms of physical activity levels needs to be comparable, and not necessarily the measurement period or the context of the activity measured. Beldo and colleagues [27] assessed the criterion validity of SGPALS against accelerometer measures, using data from wave 1 of the Fit Futures study. They concluded that SGPALS has acceptable ranking validity, thus enabling a comparison between SGPALS and among other things MVPA measured by accelerometer. The other self-reported physical activity variables have not been validated against accelerometer data.

### Outcome variable

*Symptoms of anxiety or depression* were measured at T1 and T2 by using the Hopkins Symptom Checklist-10 (SCL-10) [53], consisting of 10 items of which 6 measure symptoms of depression and 4 measure symptoms of anxiety the previous week. SCL-10 has been found to reliably measure symptoms among adolescents [54]. The 10 items were mean scored, with a range of responses between 1 and 4, in which higher values indicate more symptoms of anxiety or depression. The term mental distress, a commonly used term for self-reported symptoms of anxiety or depression, is hereafter used.

### Covariates

*Demographic variables (T1)*: Sex was coded 0 for women and 1 for men, and age at screening was numerically reported. Maternal or paternal fulltime work was controlled for in the analyses.

*Health and lifestyle covariates (T1)*: Height and weight were measured at the research site and BMI was calculated by the formula BMI = Weight (kg)/Height(m)^2^. Smoking was measured by a single item asking about present-day smoking, with response categories “daily”, “sometimes” and “no, never”. Three items measuring frequency of alcohol use, amount of alcohol normally consumed when drinking, and frequency of binge drinking during a 12-month period, were included in the questionnaire. Each item was z-scaled and subsequently summed into a composite measure. The respondents were also asked if they experienced chronic or recurrent pain lasting for 3 months or more (yes/no).

*Peer acceptance* (T1) was measured by using a subscale of the revised version of the Self-Perception Profile for Adolescents (SPPA). SPPA was originally developed by Harter [55], and revised by Wichstrøm [56] in order to be suitable for use among Norwegian adolescents. The Peer Acceptance subscale of the SPPA consists of five questions, asking if the participant finds it hard to make friends, have many friends, feel accepted among his/her peers, feel liked by peers, and feel popular among peers. Responses were given on four-point Likert scales, ranging from highly correct to highly incorrect. Negatively worded items were reversed, and a mean score variable was created.

### Statistical analyses

All analyses were carried out using the statistical software IBM SPSS Statistics for Window, version 26 (IBM Corp., Armonk, N.Y., USA). Descriptive statistics showing frequencies, means and standard deviations of the focal predictor variables, outcome and covariate variables were run. Spearman rank order correlations (for ordinal variables) or Pearson product moment correlations (for continuous variables) were run on all variables included in the main analyses. In order to see if the relationships between physical activity and mental distress among adolescents depend upon measurement method, multiple linear regression analyses were run. The focal predictor variables (four self-reported physical activity variables and two device-based variables) were investigated separately. In order to see how covariate inclusion affected the relationship between physical activity and mental distress, covariates were added in a hierarchical manner. In the first model of the cross-sectional analyses, the physical activity variable was entered together with the demographic variables (sex, age, full time work of mother and father). In model 2, health (BMI, chronic pain) and lifestyle (smoking, alcohol use) variables were added to the model, and in the final model peer acceptance was entered as a covariate. The same procedure was followed for the longitudinal analyses, only that baseline mental distress was added in the first model, alongside the predictor in question and the demographic variables. The same sample was used in the cross-sectional and longitudinal analyses.

### Treatment of missing values

In order to increase the statistical power to detect an effect and reduce possible bias, the variables, in the model with missing values (except sex and age) were imputed by using multiple imputations (MI). However, prior to imputations participants with missing values on the self-reported physical activity variables, who had reported to not be physically active outside of school on an initial question, were recoded as not active on the subsequent physical activity variables. Next, a predictive model consisting of all the variables in the dataset, also auxiliary variables not included in the analyzed models (e.g. use of medication, self-reported depression symptoms, diet, enjoyment of or barriers against physical activity and self-reported physical activity variables), was used to create 20 imputed datasets that were merged and analyzed. Variables with a high percentage of missing values (> 50%) were not imputed. In total, 449 participants had complete data on the variables included in the model. After multiple imputations, the sample size increased to 699, after selection of participants that had participated at both T1 and T2. The proportion with missing values on the variables that were imputed ranged from 12.9 to 47.8%. The variables with the highest levels of missing data were the ActiGraph data (47.8%) and T2 symptoms of mental distress (27.9%). Sensitivity analyses were performed using data from participants with complete data on all variables included in the analyses, and the results are presented below.

## Results

### Descriptive statistics

Descriptive statistics of the complete case sample and the pooled estimates of the MI-sample are shown in Tables 1 and 2. Only descriptive statistics of the complete case sample will be commented on, as MI may provide ambiguous point estimates in descriptive statistics. 60.4% of the sample were female. For frequency of physical activity, never or rarely being physically active (36.7%) was the most frequently reported response, but regarding time spent being physically active outside of school, the highest category, 4 to 7 h of physical activity, was the most common response. The majority of the adolescents reported being sedentary (13.8%) or doing mainly low intensity physical activity (36.5%) during leisure time. Mean level of minutes in LPA per valid day was 311.64, which corresponds to more than 5 h per valid day. The mean level of minutes in MVPA per valid day was 56.14. Mean level of mental distress at baseline was 1.50 (standard deviation (SD) = 0.53), and at follow-up 1.57 (SD = 0.60).

**Table 1:** Descriptive statistics of the original sample with complete data on the baseline variables used in the present study and the imputed sample

	Original sample (*N* = 449)		Imputed sample (*N* = 699)	
	*N*	%	*N*	%
Female	271	60.4	383	54.9
Male	178	39.6	316	45.1
Mother not fulltime work	135	30.1	211	30.2
Mother fulltime work	314	69.9	488	69.8
Father not fulltime work	81	18.0	132	18.9
Father fulltime work	368	82.0	567	81.1
Frequency of PA^a^				
Never or rarely	165	36.7	268	38.3
2–3 days per week	162	36.1	245	35.1
4 or more times per week	122	27.2	186	26.6
Hours of PA^a^ per week				
No or 30 min	129	28.7	222	31.8
1–3 h per week	129	28.7	184	26.3
4 to 7 h per week	191	42.6	293	41.9
Intensity of PA^a^				
No exercise/not hard	130	29.0	220	31.5
Somewhat to quite hard	184	41.0	279	40.0
Very to extremely hard	135	30.0	200	28.5
Leisure time PA^a^				
Inactive	62	13.8	135	19.3
Some light PA	164	36.5	234	33.5
Regular PA	131	29.2	188	26.9
Regular hard PA	92	20.5	142	20.3
Does not smoke	382	85.1	564	80.7
Smokes sometimes	56	12.5	111	15.9
Smokes daily	11	2.4	24	3.4
No chronic pain	338	75.3	528	75.5
Chronic pain	111	24.7	171	24.5

^a^*PA*,physical activity

**Table 2:** Mean and standard deviation of the non-categorical variables

	Original sample (*N* = 449)		Imputed sample (*N* = 699)	
	*M*	*SD*	*M*	*SD*
Age^a^	16.20	0.82	16.26	0.98
LPA^b^	311.64	64.50	311.60	80.76
MVPA^c^	56.14	24.38	56.96	22.37
Mental distress T1^d^	1.50	0.53	1.51	0.54
Mental distress T2^d^	1.57	0.60	1.57	0.60
BMI^a^	22.22	3.89	22.31	3.99
Alcohol use^e^	5.58	3.41	5.56	3.69
Peer acceptance^d^	3.27	0.49	3.31	0.48

^a^Covariates were measured at baseline

^b^*LPA*, light physical activity

^c^*MVPA*, Moderate or vigorous physical activity

^d^Range 1-4

^e^Range 1-3

### Correlations between variables included in the main analyses

The self-reported physical activity variables were highly positively correlated with each other (*r*_*s*_ between 0.60 and 0.84), whereas the positive correlation between the self-reported and objectively measured MVPA were low to moderate (*r*_*s*_ between 0.21 and 0.31) and non-significant between self-reported physical activity and LPA. There was a significant negative correlation between the physical activity variables and mental distress at baseline (*r*_*s*_ between −0.12 and −0.20) in all cases but LPA. The strength of the correlations between baseline physical activity and mental distress at follow-up were lower, and significant only for frequency of weekly physical activity, hours of physical activity and leisure time physical activity. All physical activity variables were significantly correlated with peer acceptance (*r*_*s*_ or *r* between 0.10 and 0.28) whereas mental distress had a stronger correlation with peer acceptance (*r* = −.36 at baseline, and *r* = −.26 at follow-up). The correlations are presented in Table 3.

**Table 3:** Spearman’s rank order correlations and pearson product moment correlations between all variables included in the analyses. *N* = 699

	1	2	3	4	5	6	7	8	9	10	11	12
1. Frequency of weekly PA^a^	1											
2. Hours of weekly PA^a^	0.84**	1										
3. Intensity of PA^a^	0.73**	0.77**	1									
4. Leisure time PA^a^	0.72**	0.70**	0.60**	1								
5. LPA^b^	0.08	0.05	0.02	0.08	1							
6. MVPA^b^	0.31**	0.29**	0.21**	0.27**	0.19**	1						
7. Age^b^	− 0.00	− 0.05	− 0.05	0.00	0.05	− 0.07	1					
8. Mental distress T1^b^	− 0.19**	− 0.16**	− 0.14**	− 0.20**	− 0.05	− 0.12**	0.14**	1				
9. Mental distress T2^b^	− 0.11**	− 0.08*	− 0.05	− 0.15**	− 0.06	0.05	0.06	0.58**	1			
10. BMI^b^	0.01	0.00	− 0.01	− 0.01	0.01	0.03	11**	0.04	0.01	1		
11. Alcohol use^b^	− 0.08*	− 0.04	− 0.03	− 0.04	0.02	− 0.05	0.02	0.12**	0.06	0.01	1	
12. Peer acceptance	0.24**	0.25**	0.27**	0.28**	0.10*	0.11**	− 0.13**	− 0.36**	− 0.26**	− 0.06	0.20**	1

^a^Spearman´s rank order correlations

^b^Correlation coefficients between self-reported PA variables and continuous variables are Spearman´s rank order correlations, correlations between continuous variables are Pearson product moment correlations

*p ≤.05

**p ≤.01

***p ≤.001

### Mental distress and self-reported measurements of physical activity

The results of the regression analyses of the cross-sectional relationship between self-reported frequency of physical activity and mental distress showed that never or rarely being physically active was related to more mental distress compared to the reference group (when active 4 or more times per week) (see Table 4). This was true when adjusting for demographic factors (model 1: *B* = 0.23, *p* <.001), health and lifestyle factors (model 2: *B =* 0.21, *p* <.001) and peer acceptance (model 3: *B* = 0.10, *p* =.029). The same pattern was observed for participants active 2–3 times per week although with somewhat lower effect sizes (model 1: *B* = 0.14, *p* =.004, model 2: *B* = 0.15, *p* =.002, model 3: *B* = 0.10, *p* =.033). Doing none or up to half an hour of physical activity per week was associated with higher mental distress compared to the reference group, being physically active for 4 or more hours per week, when adjusting for demographic factors (model 1: *B* = 0.17, *p* <.001) and health and lifestyle factors (model 2: *B* = 0.15, *p* =.001). Adjusting for peer acceptance in model 3 rendered the effect non-significant. Doing 1–3 h of activity per week was not significantly different from the reference group. The analyses of the relationship between self-reported intensity of physical activity and mental distress showed that no exercise or no hard physical activity compared to very to extremely hard physical activity was related to higher mental distress when adjusting for demographic characteristics (model 1: *B* = 0.16, *p* =.002) and health and lifestyle factors (model 2: *B* = 0.14, *p* =.005). Adjusting for peer acceptance gave a non-significant effect. A similar pattern was evident from the analyses of self-reported leisure time physical activity on mental distress. Being mainly inactive in leisure time was related to more mental distress compared to regular hard leisure time physical activity when adjusting for demographic factors (model 1: *B* = 0.30, *p* <.001), health and lifestyle factors (model 2: *B* = 0.28, *p* <.001) and peer acceptance (model 3: *B* = 0.13, *p* =.020). Also, doing some light physical activity in leisure time was related to higher mental distress when adjusting for demographic characteristics (model 1: *B* = 0.18, *p* =.001) and health and lifestyle factors (model 2: *B* = 0.18, *p* =.001), compared to the reference group doing hard physical activity. Including peer acceptance in the final model rendered the effect non-significant.

**Table 4:** Hierarchical linear regression models of the cross-sectional relationship between self-reported and device based physical activity measurements and symptoms of anxiety or depression at baseline. *N* = 699

Self-reported **PA**^**d**^	Model 1^a^		Model 2^b^		Model 3^c^	
	*B (95% CI)*	*p*	*B (95% CI)*	*p*	*B (95% CI)*	*p*
Frequency of weekly PA^d^ PA^d^						
Never or rarely	0.23 (0.13, 0.32)	< 0.001	0.21 (0.12, 0.30)	< 0.001	0.10 (0.01, 0.19)	0.029
2–3 times	0.14 (0.05, 0.24)	0.004	0.15 (0.05, 0.24)	0.002	0.10 (0.01, 0.18)	0.033
4 or more times^e^						
Hours of weekly PA^d^						
0 to half an hour	0.17 (0.08, 0.26)	< 0.001	0.15 (0.07, 0.24)	0.001	0.05 (−0.04, 0.13)	0.252
1 to 3 h	0.07 (−0.02, 0.17)	0.145	0.08 (−0.01,0.17)	0.084	0.04 (−0.05, 0.12)	0.360
4 or more^e^						
Intensity of PA^d^						
No exercise/not hard	0.16 (0.06, 0.26)	0.002	0.14 (0.04, 0.23)	0.005	0.01 (−0.08, 0.11)	0.775
A bit to quite hard	0.02 (−0.07, 0.12)	0.614	0.02 (−0.07, 0.11)	0.668	−0.04 (−0.13, 0.04)	0.331
Very hard^e^						
Leisure time PA^d^						
Inactive	0.30 (0.18, 0.42)	< 0.001	0.28 (0.16, 0.39)	< 0.001	0.13 (0.02, 0.25)	0.020
Some light PA	0.18 (0.08, 0.29)	0.001	0.18 (0.07, 0.28)	0.001	0.08 (−0.02, 0.18)	0.120
Regular PA	0.06 (−0.05, 0.17)	0.294	0.08 (−0.02, 0.19)	0.126	0.03 (−0.07, 0.13)	0.529
Regular hard PA intensity^e^						
Device-based measurements						
Minutes in LPA^f^	−0.01 (−0.02, −0.00)	0.044	−0.01 (−0.02, −0.00)	0.022	−0.01 (−0.02, 0.00)	0.170
Minutes in MVPA^g^	−0.03 (−0.06, −0.00)	0.026	−0.03 (−0.06, −0.01)	0.018	−0.002 (−0.04, −0.01)	0.160

^a^Model 1 includes the covariates sex, age, maternal and paternal fulltime work

^b^Model 2 includes all covariates from model 1 + BMI, smoking, chronic pain and alcohol use

^c^Model 3 includes all covariates from model 1 and 2 + peer acceptance

^d^*PA, *physical activity

^e^Reference group

^f^*LPA, *light physical activity, in 15 minutes increments

^g^*MVPA*, Moderate or vigorous physical activity in 15 minutes increments

### Mental distress and device-based measurements of physical activity

The results of the hierarchical linear regression analyses of the association between objectively measured physical activity and mental distress showed that more minutes in LPA was significantly related to lower levels of mental distress when adjusting for demographic variables (model 1: *B* = −0.01, *p* =.044) and health and lifestyle factors (model 2: *B* = −0.01, *p* =.022). A similar pattern was evident in the analyses of the association between minutes in MVPA and mental distress; more minutes in MVPA was related to lower mental distress (model 1: *B* = −0.03, *p* =.026, model 2: *B* = −0.03, *p* =.018). Adjusting for peer acceptance in the final model rendered the associations non-significant. The results of the cross-sectional models may be seen in Table 4.

### Longitudinal associations between physical activity and mental distress

None of the self-reported or device based physical activity variables measured at baseline were related to higher risk of mental distress at follow-up, after adjustment for baseline mental distress. The results of the longitudinal analyses are displayed in Table 5.

**Table 5:** Hierarchical linear regression models of the longitudinal relationship between baseline self-reported and device based physical activity measurements and symptoms of anxiety or depression at follow up. *N* = 699

Self-reported PA ^d^	Model 1^a^		Model 2^b^		Model 3^c^	
	*B (95% CI)*	*p*	*B (95% CI)*	*p*	*B (95% CI)*	*p*
Frequency of weekly PA^d^						
Never or rarely	−0.01 (−0.10, 0.09)	0.908	0.02 (−0.07, 0.12)	0.633	0.01 (−0.09, 0.10)	0.910
2–3 times	−0.01 (−0.10 0.08)	0.852	0.01 (−0.09, 0.10)	0.915	−0.02 (−0.10, 0.09) 1.93)	0.974
4 or more times^e^						
Hours of weekly PA^d^						
0 to half an hour	−0.02 (−0.11, 0.07)	0.637	0.00 (−0.08, 0.09)	0.922	0.01 (−0.10, 0.08)	0.766
1 to 3 h	0.01 (−0.09, 0.10)	0.915	0.01 (−0.08, 0.10)	0.704	−0.01 (−0.08, 0.10)	0.808
4 or more^e^						
Intensity of PA^d^						
No exercise/not hard intensity	−0.07 (−0.16, 0.02)	0.147	−0.05 (−0.15, 0.04)	0.287	−0.08 (−0.17, 0.02)	0.122
A bit to quite hard	−0.07 (−0.15, 0.02)	0.142	−0.07 (−0.15, 0.02)	0.142	−0.08 (−0.17, 0.01)	0.077
Very hard^e^						
Leisure time PA^d^						
Inactive	0.03 (−0.09, 0.15)	0.573	0.06 (−0.05, 0.18)	0.292	0.04 (−0.08, 0.16)	0.476
Some light PA^d^	−0.00 (−0.11, 0.10)	0.964	0.02 (−0.08, 0.13)	0.660	0.01 (−0.10, 0.12)	0.859
Regular PA^d^	−0.10 (−0.20, 0.01)	0.072	−0.08 (−0.19, 0.03)	0.140	−0.09 (−0.19, 0.02)	0.109
Regular hard PA^d, e^						
Device-based measurements						
Minutes in LPA^f^	−0.01 (−0.02, 0.00)	0.199	−0.01 (−0.02, 0.00)	0.183	−0.01 (−0.02, 0.00)	0.228
Minutes in MVPA^g^	0.00 (−0.02, 0.03)	0.456	0.01 (−0.02, 0.03)	0.594	0.01 (−0.02, 0.03)	0.494

^a^ Model 1 includes the covariates sex, age, maternal and paternal fulltime work, and baseline symptoms of anxiety or depression

^b^ Model 2 includes all covariates from model 1 + BMI, smoking, chronic pain and alcohol use

^c^ Model 3 includes all covariates from model 1 and 2 + peer acceptance

^d^*PA*, physical activity

^e^ Reference group

^f^*LPA*, Light physical activity in 15 min increments

^g^*MVPA*, Moderate or vigorous physical activity in 15 min increments

### Sensitivity analyses

Only 449 individuals had complete data on the variables used in the present study. As evident from Tables 1 and 2, the frequency distributions, means and standard deviations of the complete case and the multiple imputed samples were largely similar. The results of the analyses run on the complete case sample differed from the results of the analyses of the MI-sample in a few cases. In the cross-sectional analyses based on the complete sample, mental distress did not significantly differ between the group never or rarely being physically active and the reference group (being physically active 4 or more times per week) in the final model (*B* = 0.08, *p* =.158), and being physically active 2–3 times per week was not significantly different from the reference group in model 2 (*B* = 0.12, *p* =.038). Being active for 1–3 h per week was significantly different from the reference group in model 2 in the complete case sample (*B* = 0.12, *p* =.038), but not in the MI-sample. Being mainly inactive was not significantly different from the reference group (regular hard physical training and competitive sports) in model 3 in the complete case sample (*B* = 0.14, *p* =.077). The three effects that were non-significant in the complete case sample, but significant in the MI sample may be explained by an increase in statistical power to detect an effect after multiple imputation.

## Discussion

The main aim of the present study was to examine the relationship between physical activity and symptoms of anxiety or depression among adolescents in a cross-sectional and longitudinal sample, and a secondary aim was to examine the degree to which measurement method of physical activity is of importance. The results indicate that physical activity and mental distress are cross-sectionally related, up until inclusion of peer acceptance as a covariate. The results did not differ according to manner of measurement of physical activity. When adjusting for peer acceptance, only three variables remained significantly associated with mental distress; never or rarely being physically active, being active 2–3 times per week and mainly being inactive during leisure time were all related to higher mental distress when compared to the respective reference groups. In the longitudinal analyses, baseline levels of self-reported or objectively measured physical activity was not significantly related to mental distress at follow-up, when adjusted for baseline mental distress.

The degree to which one may conclude regarding a cross-sectional relationship between physical activity and mental distress depends upon the role played by peer acceptance included in the final model. The fact that adjusting for peer acceptance had such a consistent impact on the results; by reducing the variability in mental distress in previous models attributed to physical activity to a non-significant effect, indicates that the social environment of adolescents plays a substantial part in influencing mental distress. In terms of how to interpret the results, it is important to understand the role played by peer acceptance in this relationship. In particular, it is important to understand if peer acceptance plays a part in a causal chain as a mediator, or if peer acceptance acts as a confounding factor. Participating in organized sports provides an arena for impacting both physical activity levels [57] and peer relations [58]. Peer acceptance is an important predictor of sports continuation, enjoyment and motivation for activity and perceived competence in sports [42, 59, 60], which in turn are related to higher levels of physical activity [43, 61]. Having poor relationships to peers is related to depression, in a bidirectional fashion, meaning that depression predicts interpersonal problems and a deterioration of peer relations [44], and that poor peer relations have been found to predict depression [62]. Thus, it is evident that peer relations, in this study operationalized as peer acceptance, plays an important part, and that it may play a part in a causal chain between physical activity and mental distress, as a mediating variable. If this is the case, the reduction in effect of physical activity on mental distress in the final model in the cross-sectional analyses is explained by an interrelationship between physical activity, peer acceptance and mental distress. If peer acceptance is an intermediate, mediating factor, preventive efforts may be directed against enhancing the degree to which the individual feels accepted by their peers, in order to influence mental distress, rather than just focusing on increasing physical activity levels.

However, peer acceptance may also act as a confounder. As shown in the study by Daniels et al. [63], participating in organized sports may influence peer acceptance among adolescents, which in turn may be related to higher levels of physical activity [64]. Physical competence or athleticism acquired through being physically active is also related to peer acceptance [65, 66]. Thus, it may not be the activity per se that influences peer acceptance, but rather the social environment in which the majority of the physical activity is carried out or the skills acquired through being physically active. If peer acceptance acts as a confounding factor, the significant relationships identified in models 1 and 2, when peer acceptance was omitted, may represent spurious associations. However, the large body of research on the topic does not support this notion of a spurious relationship. The present study is not suitable for investigating the degree to which peer acceptance acts as a mediator or a confounding factor, as data was collected only at two time points, but future research ought to examine the interplay between the three factors in order to enhance the understanding of how physical activity impacts mental health, possibly partly through peer relationships.

While our findings indicate that the role played by peer acceptance is unclear, other studies have omitted peer acceptance or other variables measuring related social concepts, which limits direct comparison with previous findings. However, at least one study has included related variables. Babiss et al. [67] investigated the degree to which sports participation was related to depression, and found the inclusion of self-esteem and social support, in which social acceptance by peers was one variable, to attenuate the odds of depression considerably, while still remaining significant. This finding is comparable to the findings of the present study, in which the effects that were statistically significant after adjusting for peer acceptance all showed that the groups with lower physical activity levels tended to have higher mental distress compared to the reference groups with the highest levels of physical activity. Although Babiss et al. [67] state that social support partially mediates the relationship between physical activity and depression, the design of the study was cross-sectional, and as such, not suitable for investigating mediation. Thus, the role played by social acceptance in the relationship between physical activity and mental distress is still unclear and needs to be further studied.

In the present study, the longitudinal data was analyzed by investigating the association between baseline self-reported or device-based measurements of physical activity and mental distress two years later, after adjusting for baseline mental distress. The results of the analyses showed that physical activity at baseline was not significantly related to mental distress at follow-up, regardless of manner of measurement. The results are in line with a number of previous findings, as studies using self-reported or more objectively measured physical activity have often not been able to find a significant relationship with mental distress measured over time (e.g. 18, 22, 23, 29). A number of factors may have impacted mental distress between T1 and T2, and many factors are likely to be more important in predicting mental distress than physical activity. Among these are alcohol use, smoking, BMI and peer acceptance [68], which we were able to adjust for in the analyses. Nonetheless, there may have been other factors that may have impacted the outcome between baseline and follow-up. Also, physical activity levels may have changed between measurement points, and one may expect the physical activity level most proximal in time to have a greater impact on symptoms of anxiety or depression. However, a study based on the same data as the present study, found that change in objectively measured physical activity between baseline and follow-up was unrelated to change in mental distress [23]. Thus, it is unlikely that changes in physical activity levels have impacted the results greatly.

Adjusting for baseline values of a variable is common in studies with two time points, but there are some potential problems with this model, as stated in Lord’s paradox. Pearl [69] argues that adjusting for baseline values in a regression model may be understood in a mediation framework, which in this case would mean that baseline physical activity is a causal predictor of baseline mental distress, which in turn is a causal predictor of mental distress at follow-up. The direct effect of physical activity thus entails the effect of physical activity on mental distress at follow-up, when baseline mental distress is held constant. For this interpretation to be valid, the causal relationships of the mediation model need to be established, either statistically or empirically in other studies. The stability in depressive and anxiety symptoms clearly indicate that baseline mental distress is a causal predictor of mental distress at follow-up [70]. On the other hand, baseline physical activity may impact baseline mental distress, but the causal pathways may also be reversed. However, a recent study using Mendelian randomization found a protective effect of objectively measured physical activity on major depressive disorder, but not evidence of the reversed causal pathway [7]. Other methodically sound studies have also shown similar findings [71]. Thus, it is more likely that baseline physical activity is a causal factor for baseline mental distress, than the other way around, hence providing support for our chosen model.

As mentioned in the methods section, the self-reported items measured leisure time physical activity during the past year, whereas the device-based measurements assessed physical activity in both school and leisure time during a one-week period. Thus, both the timeframes and the context of the measurements differ. For a comparison of the associations between device-based and self-reported physical activity and mental distress to be constructive, the measures need to be comparable in terms of the ranking of the participants, and not necessarily in terms of similarity of construct. The study by Beldo and colleagues [27] confirms that SGPALS has satisfactory ranking validity compared to MVPA measured by accelerometer. Each increase in level of physical activity on the SGPALS corresponds to 8 more minutes of MVPA per day on average, summing up to approximately 60 min more per week per additional level on SGPALS. The correlations between SGPALS and the other self-reported measurements were high or moderately high, which most likely means that these also are comparable to MVPA measured by accelerometer. The correlations between MVPA and SGPALS, as well as with the other self-reported physical activity variables, is considerably higher compared to the correlations between LPA and all the self-reported variables. Thus, it is more questionable if LPA and the self-reported physical activity variables are comparable in terms of ranking. Individuals with high LPA do not necessarily exercise frequently or with high intensity, and as such, LPA represents a different kind of physical activity compared to the other physical activity measures. However, the statistical relationship between LPA and mental distress closely resembles the relationship between MVPA and mental distress, which indicates that physical activity, whether light or moderate/vigorous, has a significant relationship with mental distress, up until adjustment of peer acceptance in the model.

### Methodological considerations

The main strength of the present study is the use of both self-reported and more objective measurements of physical activity. Device-based measurements are superior to using self-reported physical activity, as adolescents tend to over-report time spent in moderate and vigorous activity [26], but the ranking of participants in terms of self-reported physical activity is most likely reliable [27]. The opportunity to examine if the relationship between physical activity and mental distress depends upon measurement method offers a unique chance to provide more credibility to the results of the vast amount of the literature on the topic to date. There are, however, validity issues also with device based physical activity measurements, relating to specific activities that tend to be underreported (e.g. cycling, rowing, swimming), as well as the potential of a change in behavior as a result of the behavior being measured. A measurement period of 8 days, as in the present study, provides reliable data [72], but the degree to which the physical activity performed during a short period could be expected to be related to mental distress two years later may be questionable, mainly depending upon if the physical activity levels were representative of a typical physical activity level of the individual. The physical activity performed during the measurement period may have been both higher and lower than usual. The awareness of wearing an accelerometer was not found to impact the physical activity pattern of adolescent participants in a randomized controlled trial [73], but other factors may have impacted physical activity levels, such as temporary sickness or injuries. Also, physical activity levels may have changed between T1 and T2. However, in a study based on the same data, Opdal et al. [74] showed that MVPA declined by 8.19 min between Fit Futures 1 and 2, with a standard deviation of 25.33. This mean change corresponds with the weighted mean difference of MVPA measured by accelerometer found in a meta-analysis of change in physical activity when transitioning to adulthood [75], and as such is no more than expected due to age effects. Nonetheless, the results should be interpreted with the limitation of the short measurement period in mind.

The response rate suggests high generalizability of the sample. The multiple imputed sample analyzed in the linear regression analyses constituted 67.3% of the sample participating at baseline (92.9% of the invited sample), which is considered high in population-based samples.

There are also some limitations that need to be considered. A substantial number of participants did not have valid accelerometer data. This may be due to several reasons, ranging from lack of adherence to the protocol (i.e., not compliant with at least 4 days with 10 h of data) to a decline of the invitation to participate in the accelerometer study. Potentially, missing values may introduce bias in the results, depending on the degree to which the missing values are random or systematic, and dependent upon observed or unobserved data. Data that is missing completely at random (MCAR) or at random (MAR) can be handled well by multiple imputation. If missing values are not MAR, both the usage of multiple imputation and complete case analyses will provide biased results. The problem is that the degree to which values are MAR is most often not known, without conducting follow-up studies of the participants with missing data. Multiple imputation is considered to lower the risk of bias and increase statistical power [76], and was deemed necessary. Comparing the sample with missing data (that was subsequently imputed) to the sample without missing data may provide some information regarding the precision of the imputation, and the sensitivity analyses and the frequency distributions, means and standard deviations presented in the descriptive statistics showed no systematic differences. However, this does not guarantee that data is MAR, as missingness may depend upon unobserved variables. In order to increase the chance of non-biased estimates, we included variables that may be predictive of missing values in the imputation model [77]. Using multiple imputation, we have lowered the risk of significant bias due to missing values, but the risk of bias is still present. Collecting self-reported data on physical activity should be considered as a precaution in studies using accelerometer data, where the risk of missing data is high.

Mental distress was measured by self-report, which may be prone to information bias [78]. Parental full-time work was used as a proxy to socio-economic status in the analyses. This is obviously a limitation, and as such it is uncertain if socioeconomic status has been adequately adjusted for.

As the sample was selected from two municipalities in northern Norway, it is not evident whether the results are generalizable to the Norwegian population of adolescents as a whole. Generalizability was enhanced by recruitment of one urban and one rural municipality. The objective measurements of physical activity were conducted during winter for more than 80% of the sample, and thus, it is unlikely that season of measurement has impacted the results. However, it is possible that the variability in the objective physical activity measurements was lower as a result. Variability in measurements may impact the statistical power to detect an effect, and as such, a greater variability in time at which the accelerometer was worn may have had an impact.

## Conclusion

The results indicate that physical activity and mental distress are related, when measured cross-sectionally, up until inclusion of peer acceptance as a covariate. This finding was independent of manner of measurement. This means that the social environment of adolescents is of importance, although the present study is unable to examine how peer acceptance impacts the relationship between physical activity and mental distress. If the social environment plays a causal part in the relationship between physical activity and mental distress, interventions may incorporate this aspect into the program. Physical activity at baseline was not significantly related to mental distress two years later when baseline mental distress was adjusted for. Future studies investigating the relationship between physical activity and mental distress need to take into account, and examine in detail, how the social environment of the adolescent, impacts the relationship. This may be imperative in order to understand how the mental health of adolescents may be enhanced, either directly or indirectly through being physically active.

## Data Availability

Data from the Fit futures study may be obtained from a third party, UiT – The Artic University of Norway. Restrictions apply to the availability of these data, which were used under license for the current study, and are thus not publicly available. Access may be applied for by contacting fitfutures@uit.no.
